# Modeling HIV/AIDS Drug Price Determinants in Brazil: Is Generic Competition a Myth?

**DOI:** 10.1371/journal.pone.0023478

**Published:** 2011-08-15

**Authors:** Constance Meiners, Luis Sagaon-Teyssier, Lia Hasenclever, Jean-Paul Moatti

**Affiliations:** 1 Economic and Social Sciences, Health Systems and Society (SE4S), UMR 912 (INSERM, IRD, Aix-Marseille University), Marseille, France; 2 Institute of Economics, Federal University of Rio de Janeiro, Rio de Janeiro, Brazil; 3 Ministry of Planning, Budget and Administration, Brasilia, Brazil; 4 ORS-PACA (Regional Observatory for Disease Control in South-Eastern France), Marseille, France; University of Oxford, Viet Nam

## Abstract

**Background:**

Brazil became the first developing country to guarantee free and universal access to HIV/AIDS treatment, with antiretroviral drugs (ARVs) being delivered to nearly 190,000 patients. The analysis of ARV price evolution and market dynamics in Brazil can help anticipate issues soon to afflict other developing countries, as the 2010 revision of the World Health Organization guidelines shifts demand towards more expensive treatments, and, at the same time, current evolution of international legislation and trade agreements on intellectual property rights may reduce availability of generic drugs for HIV care.

**Methods and Findings:**

Our analyses are based on effective prices paid for ARV procurement in Brazil between 1996 and 2009. Data panel structure was exploited to gather ex-ante and ex-post information and address various sources of statistical bias. In-difference estimation offered in-depth information on ARV market characteristics which significantly influence prices. Although overall ARV prices follow a declining trend, changing characteristics in the generic segment help explain recent increase in generic ARV prices. Our results show that generic suppliers are more likely to respond to factors influencing demand size and market competition, while originator suppliers tend to set prices strategically to offset compulsory licensing threats and generic competition.

**Significance:**

In order to guarantee the long term sustainability of access to antiretroviral treatment, our findings highlight the importance of preserving and stimulating generic market dynamics to sustain developing countries' bargaining power in price negotiations undertaken with originator companies.

## Introduction

Brazil became the first developing country to guarantee free and universal access to Highly Active Antiretroviral Therapy (HAART). Access to HIV/AIDS treatment was established as a legal right in 1996, but public delivery of antiretroviral drugs (ARVs) started as early as 1991. In 2009, HAART was delivered to nearly 190,000 people living with HIV and AIDS (PLWHA), covering more than 90% of estimated need according to previous 2006 World Health Organization (WHO) guidelines [1]. Access to HIV/AIDS treatment in Brazil has been sustained through a set of strategies mixing local generic production of off-patent ARVs, centralized procurement, and the threat of issuing compulsory licenses on patent-protected drugs [2]. Historically, Brazil's defiance to originator company monopolies produced positive spillovers to antiretroviral treatment (ART) scaling-up programs in other developing countries. Brazilian imports of active pharmaceutical ingredients (APIs) from countries such as India and China facilitated the creation of an international market for generic ARVs [3]. From 1998 to 2004, there was a 2.5-fold increase in the number of treated patients while mean annual ARV spending per patient in Brazil, including all patient groups (prophylactic, pediatric, and adult) and regimens, decreased more than 73%. This trend, however, has been interrupted since 2005, due to both the incorporation of new ARVs to tackle therapeutic toxicity and drug resistance and the need of a growing number of patients to move on to more expensive second and third-line regimens. In 2009, according to information provided by the Brazilian Ministry of Health, total ARV spending approached $316 million, with nearly 72.5% of this budget spent on drugs delivered exclusively by originator patent-holding companies.

The Brazilian experience in ART provision and its drawbacks can help anticipate many of the issues soon to afflict other developing countries, as the 2010 revision of the WHO guidelines shifts demand towards more expensive therapies [4], [5]; at the same time, current evolution of international legislation and trade agreements on intellectual property rights (IPRs) may reduce availability of generic drugs for HIV care [6]–[8]. As Table 1 shows, the Brazilian Health System provides the decentralized delivery of 20 ARVs and one fixed dose combination (FDC), totaling 33 child and adult formulations. Pharmaceutical patent protection has been enforced in the country since May 1997. Considering the latest available information on patent approvals and pending patent applications in Brazil, from the Brazilian Institute of Intellectual Property patent database, nine of the ARV drugs included in Table 1 are under IPR protection. The patent on Abacavir expired in June 2008. That same year, the patent on Tenofovir was denied. Regarding the use of compulsory licenses, although several threats to grant them were made in the past, notably involving drugs such as Nelfinavir, Efavirenz and Lopinavir boosted by Ritonavir, only one compulsory license was actually declared on Efavirenz in May 2007 [2].

**Table 1:** ARV Drugs Delivered in Brazil.

Therapeutic Class	Generic Name	Inclusion Year
Nucleoside Reverse Transcriptase Inhibitor (NRTI)	Zidovudine (AZT)*	1991
	Didanosine (ddI)* ¤	1993
	Zalcitabine (ddC)×	1996
	Lamivudine (3TC)*	1996
	Estavudine (d4T)*	1997
	AZT+3TC [FDC]*	1998
	Abacavir (ABC)	2001
	Tenofovir (TDF)	2003
	Didanosine EC (ddI EC)	2005
Non-Nucleoside Reverse Transcriptase Inhibitor (NNRTI)	Nevirapine (NVP)*	1998
	Delavirdine (DLV) ×	1999
	Efavirenz (EFV)* +	1999
	Etravirine (ETR)	2010
Protease Inhibitor (PI)	Saquinavir (SQV)*	1996
	Ritonavir (RTV)	1996
	Indinavir (IDV)*	1997
	Nelfinavir (NFV)+ ×	1998
	Amprenavir (APV)^+¤^	2001
	Lopinavir/RTV (LPV/r)+	2002
	Atazanavir (ATV)+	2004
	Fosamprenavir (FPV)+	2005
	Darunavir (DRV)+	2008
Fusion Inhibitor (FI)	Enfuvirtide (T-20)+	2005
Integrase Inhibitor (II)	Raltegravir (RAL)+	2009

*Locally produced ARV (2009);

+ IPR protected;

¤ Child formulation only;

× No longer available.

ARV procurement in Brazil is centralized by the Ministry of Health, which is also in charge of issuing HAART guidelines. These guidelines are discussed and established by an independent group of experts, where priority is given to treatment quality over costs [9]. Periodical revising of treatment recommendations takes place in order to incorporate the latest available medical evidence. The inclusion of new ARVs in these guidelines has sometimes been accelerated by repeated lawsuits filed by individuals and civil society groups [10]–[12]. Another important feature of the HIV care policy in Brazil regards treatment individualization. In order to maximize adherence to treatment and postpone resistance, a vast array of ARVs is available in first-line prescription formularies, allowing physicians to best adapt therapy to patient's individual characteristics and needs. Although the Brazilian government holds strong bargaining power as the sole purchaser of ARVs in the country, this power may be limited by the large freedom physicians have to prescribe the ARV regimen they judge most appropriate for their patients. These prescriptions must, however, conform to treatment guidelines, which impose strict restrictions concerning the use of drugs reserved for “salvage therapy” (as for Enfuvirtide, Darunavir, Raltegravir, and, most recently, Etravirine), for the treatment of pregnant women and in cases of co-morbidities, such as hepatitis and tuberculosis, to avoid drug interactions. [12] The existence of such restrictions, added to the fact that HAART must combine at least three drugs from two different classes, reduces the possibilities of substituting one ARV for another and favors market concentration [13].

Looking into the ARV market supply side, originator companies and generic suppliers form two quasi-parallel market segments. Among ARV manufactures registered at the Brazilian National Health Surveillance Agency in 2009, eight were public laboratories, five locally-owned private companies, eight originator companies and two foreign generic suppliers. Patented drugs are strictly supplied by foreign patent or license-holding companies. An exception applies to Efavirenz which, after compulsory licensing in 2007, has ultimately been supplied by a public laboratory. Non-patented ARVs are supplied by local and foreign companies. Local generic production, involving both public laboratories and private companies, provided eight ARVs and one FDC in 2009 (see Table 1). Additionally, local supply of generic Ritonavir took place from 2002 to 2006. As for generic Tenofovir, local supply is to begin in 2011 [14]. Finally, imports from foreign generic or originator companies for the supply of non-patented ARVs occurs when local production proves insufficient to satisfy demand.

ARV production in Brazil initiated in 1993, at a private laboratory, and was followed by the public sector in 1994. Pressured by the soaring costs of ART provision, in 1998 the government reactivated pharmaceutical production capacity in public laboratories that had been lying dormant since the previous decade [15]. Generic ARV supply has then been progressively shifted to public facilities. Since 2002, local private production of generic ARVs has specialized in the supply of drugs or formulations not provided by the public sector [13]. This led to an important decrease in the total number of generic suppliers present in the Brazilian ARV market, from 19 in 2002, down to 5 in 2009. Furthermore, considering that public laboratories' capacity is restricted to drug formulation, local ARV production is heavily dependent on API imports from China and India [13], [16]. Public API procurement policy, based on lowest price rather than quality criteria, coupled by negative tax and regulatory discrimination towards local companies, has contributed to the dismantling of private API producers in the Brazilian territory who have been unable to compete in equal terms with foreign suppliers [17].

Over the past decades pharmaceutical price regulation in Brazil underwent considerable changes. During the 1990s, pharmaceutical drugs were not subject to price control in the Brazilian market [18]. A formal regulation system was introduced in 2000 when the Drug Chamber was created as part of the Brazilian National Health Surveillance Agency. From that year until 2003, when the Drug Chamber was replaced by the Chamber of Drug Market Regulation, price control was based on retrospective manufacturing costs [19]. Since 2003, drug prices are adjusted on an annual basis according to a price cap derived from past inflation, expected sector productivity, observed pharmaceutical input cost variation and level of intraclass market concentration [20]. The price of new drugs take into account prices practiced in the international market, while generic drugs have had their prices set at, in the least, 35% lower than the price of the originator drug [19]. In the case of ARVs, however, since the Ministry of Health has a regulatory monopoly for their procurement, the usual pharmaceutical price regulation mechanism does not directly apply and ARV prices are the outcome of negotiations between the Ministry and the various suppliers.

This paper aims at empirically analyzing factors that have influenced ARV price evolution in Brazil and achieving an understanding of their effects on market dynamics. Previous studies of the Brazilian ARV market [21]–[23] have adopted descriptive approaches, while recent attempts to model ARV price determinants at the global level [24], [25], [27] have faced complex methodological problems due to lack of data standardization and limited comparability. The Global Price Reporting Mechanism, set by the WHO, and the Global Fund Price and Quality Reporting Tool databases employed by these analyses mainly contain donor-funded procurement transactions. In this sense, they may not be representative of ARV prices paid in the developing world. Additionally, their prices are not standardized according to tax, shipping and insurance supplements. Our study relies on exhaustive and standardized data covering ARV procurement in Brazil from 1996 to 2009. Considering that these data, which are provided by the Brazilian Ministry of Health, are used for budgeting and accountability procedures, their reliability is very high. Taking into account a set of candidate drug price determinants, we have employed an econometric approach adapted to the characteristics of the Brazilian ARV market structure. We further address important methodological issues concerning demand endogeneity and unobserved heterogeneity bias that, to our knowledge, have not been dealt with by previous analyses.

## Materials and Methods

### Dataset Configuration

Our analyses are based on effective prices paid for ARV procurement in Brazil between 1996 and 2009. The study dataset has been created by gathering information on transaction and market characteristics from different sources. Data on ARV transactions, including drug strength and dosage form, supplier, date, quantity and price, as well as number of treated patients have been provided by the Brazilian Ministry of Health. Data on registered ARV suppliers have been collected from the Brazilian National Health Surveillance Agency electronic database. United States (US) Food and Drug Administration (FDA) market approval dates have been included, as proxies of first launch in any market, in order to calculate drug age. Brazilian HIV/AIDS treatment guidelines, issued between 1996 and 2008, have been used to gather information on drugs allowed in first-line therapy and possible intraclass drug substitutions. Annual Gross Domestic Product (GDP) was obtained from the Central Bank of Brazil website. Finally, patent information has been checked using the Brazilian Institute of Industrial Property patent database.

For the purposes of our analysis, we have selected transactions on drugs used for adult treatment for which standard yearly dosage could be calculated. We excluded observations referring to FDCs for consistency of comparability. Our initial sample included 378 observations referring to transactions on 21 ARVs (Appendix S1). All transaction prices include insurance and freight up to delivery point; imported ARVs are delivered directly to the Brazilian Ministry of Health; locally produced ARVs may be delivered to this same organism or to state governments. No import tax applies to ARVs. All prices have been converted to US dollars, applying geometric mean exchange rates per transaction year, calculated by using daily rates provided by the Central Bank of Brazil. These prices have been adjusted for inflation to 2009 dollars using the annual US Consumer Price Index for medical care items, available from the US Bureau of Labor Statistics. Finally, the procured quantities and transaction prices have been standardized in terms of yearly dose, as defined by current Brazilian HIV/AIDS treatment guidelines:


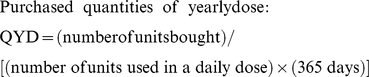



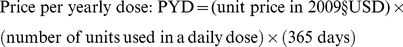


In the case of protease inhibitors which are boosted by the drug Ritonavir, their combined annual cost has been considered for calculating PYD.

We next exploited our dataset panel structure to gather ex-ante and ex-post information. Transaction pairs were constructed from observations for ARVs purchased from the same supplier in two consecutive years. This manipulated dataset resulted in 246 observations, which exhibited a structure similar to that of the original dataset (Appendix S2). This allowed keeping row-information on *t* and *t+1* transactions. For example, if AZT has been supplied by the same supplier S1 from 1996 to 2000, in our subset, AZT_S1_ will be associated to four rows. Holding supplier constant enabled us to identify changes in transaction characteristics and their consequences on price evolution.

### Analytic Approach

We carried out two analyses over the dataset, one concerning the entire market and another stratified according to originator (n = 78) and generic ARV suppliers (n = 168). We first estimated PYD determinants from the following multivariate regression equation, using pooled ordinary least squares (OLS):


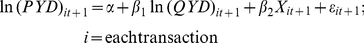
(1)

Our dependent variable, PYD, was transformed in natural logarithm. Explanatory variables were: the natural logarithm of demand quantity (QYD); ARV therapeutic class - NRTI, NNRTI, PI and FI; drug age - whether five years and more after FDA approval ( = 1) or not ( = 0); weight specific formulation - whether exclusive for adult patients weighting less than 60 kg ( = 1) or not ( = 0); therapeutic recommendations - whether included in first-line HAART recommendations ( = 1) or not ( = 0); number of intraclass substitutes; number of potential suppliers in the market - number of registered suppliers in the respective transaction year; and, type of supplier - whether originator ( = 1) or generic ( = 0). Time evolution was controlled by the introduction of two different aggregate conditions in the model: the natural logarithm of GDP, in current 2009 US dollars; and, number of treated patients. These variables were rescaled to 1:100,000 and 1:10,000 respectively. For the purpose of correcting collinearity between them, ln(GDP) was further detrended (before detrending: ρ = 0.942, p<0.001; after detrending: ρ = 0.075, p = 0.202).

In order to establish estimation consistency, we tested demand exogeneity, i.e., whether demand quantity is effectively independent of price. Conventional economic theory suggests that demand quantity (purchase volume) and price reciprocally influence each other and should be mutually explained. Including demand as a determinant of price in an econometric estimation, therefore, raises an important methodological issue called endogeneity. As it might be impossible to observe all relevant attributes of the ARV market in Brazil, and in other countries as well, ARV demand may itself reflect some of these attributes: unobserved variables such as the context of price negotiation or other characteristics pertaining to the procurement process may affect both the volumes of drugs actually purchased and the level of prices, and consequently demand quantity cannot be considered as an independent determinant of price. However, in the Brazilian context, where there is legal obligation to provide universal access to HAART free of charge, it is possible to make the hypothesis that ARV demand is defined on the basis of medical needs and independently of price. To check this exogeneity hypothesis, we implemented the augmented regression test suggested by Davidson and McKinnon (1993) [23]. Previous analyses on ARV price determinants have introduced drug purchase volume as an independent explanatory variable but, to our knowledge, they have failed to test this important assumption [24]–[26]. This may be due to difficulties in finding appropriate instruments. In our case, this has been made possible by exploiting row-data information. Our pooled OLS model has been estimated at *t+1* to allow employing lagged QYD as an instrument.

OLS regression is the most common technique used for analyzing the relationship between one or more explanatory variables and the dependent variable. The implementation of pooled OLS estimations is – undoubtedly - the best way of identifying the determinants of an outcome, such as ARV prices. Nevertheless, pooled OLS estimation makes the assumption that available information suffices to explain price variability. This may pose additional methodological problems when the dependent variable is susceptible to being affected by unobserved factors. This strong assumption can be relaxed by controlling unobserved heterogeneity. In-difference estimation using ex-ante and ex-post information allows controlling for both time-constant observed and unobserved heterogeneity. It also has the advantage of correcting variable endogeneity, since unobserved heterogeneity constitutes the main source of this problem [28], [29]. However, the implementation of this technique requires large datasets in order to construct observation-pairs that reflect changes between two points in time. Another fallback is that this technique does not provide information on the specific effect of time-constant factors although these are controlled for. For this reason, the in-difference regression is often implemented as a complement to pooled OLS estimation. In the in-difference model we use, supplier is kept constant and row-price differentials are explained by time-varying factors available in the dataset: changes in demand quantity; therapeutic recommendations; number of intra-class substitutes; number of potential suppliers; as well as GDP and number of treated patients.



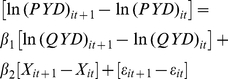
(2)


## Results

### Time-varying Characteristics

The manipulated dataset allowed for the observation of Brazilian ARV market time-varying characteristics. Table 2 illustrates mean price and quantity row-differentials over the study period. Overall, between two consecutive years, mean PYD decreased 15% and mean QYD increased 3%. Mean interannual PYD decrease has been evenly distributed between originator and generic segments (15%). Nonetheless, mean originator drug demand increased at an average rate of 21% while the demand for generic drugs decreased at nearly 5%, from one year to the next.

**Table 2:** Interannual PYD and QYD Differentials in the Brazilian ARV Market (1996–2009).

	All		Originator Drugs		Generic Drugs	
Row-Differentials	(n = 246)		(n = 78)		(n = 168)	
	Mean	sd	Mean	sd	Mean	sd
**Ln(PYD)**	−0.150	0.328	−0.148	0.205	−0.150	0.372
**Ln(QYD)**	0.032	1.151	0.206	1.455	−0.049	0.974

Table 3 shows relative frequency of ARV transaction characteristics. Nearly 85% of originator drug transactions involved a price decrease, compared to 55% of generics. QYD, on the other hand, showed near even distribution between segments. Although mean changes in guideline therapy recommendations from one year to another took effect only in a minority of cases (i.e., involving less than 17% of originator drugs and nearly 7% of generic drugs in our sample), they mostly favored including originator drugs (12% against 5% for generic drugs). As for market competition, considering the number of intraclass substitutes, product alternatives increased in about 27% of the cases. In terms of potential suppliers, which concerns mainly off-patent drugs, for every two consecutive years, more than half of generic transactions involved fewer number of competitors than the previous year.

**Table 3:** Observed Interannual Changes in Brazilian ARV Market Characteristics (1996–2009).

	All	Originator Drugs	Generic Drugs
Change between *t* and *t+1*	(n = 246)	(n = 78)	(n = 168)
	%	%	%
***PYD***			
Decrease	64.2	84.6	54.8
Increase	35.8	15.4	45.2
***QYD***			
Decrease	47.2	50.0	45.8
Increase	50.4	48.7	51.2
Without Change	2.4	1.3	3.0
***First-Line Therapy***			
Exclusion	3.3	5.1	2.4
Inclusion	6.9	11.5	4.8
Without Change	89.8	83.3	92.9
***Intraclass Substitutes***			
Decrease	14.2	16.7	13.1
Increase	26.8	25.6	27.4
Without Change	58.9	57.7	59.5
***Potential Suppliers***			
Decrease	39.0	5.1	54.8
Increase	28.1	24.4	29.7
Without Change	32.9	70.5	15.5

### Models Estimation

Pooled OLS estimation results are presented in Table 4, after correcting for QYD endogeneity present in the general market and the generic segment by applying the instrumental variable in two-stage least squares (2SLS) specification (Appendix S3). The “all” column shows estimates for the general market. The association between demand volume and price is statistically significant (p = 0.001) and can be here interpreted as partial elasticity. *Ceteris paribus,* a 1% demand increase results in close to 0.24% mean price decrease. The effect of type of supplier stands out, as originator drugs cost, on average, 132% more than generic drugs (p<0.001). Considering that the Brazilian ARV market structure is better characterized by two parallel segments (originator and generic drug supply), we next compare stratified estimations.

**Table 4:** Price Determinants in the Brazilian ARV Market (1996–2009): Pooled OLS at *t+1.*

Dependent Variable: Ln(PYD)	All		Originator Drugs		Generic Drugs	
	(n = 246)		(n = 78)		(n = 168)	
	2SLS		OLS		2SLS	
Explanatory Variables	Coeff.	SE	Coeff.	SE	Coeff.	SE
**Intercept**	7.584***	0.535	8.712***	0.358	6.476***	0.786
**Ln(QYD)**	−0.235***	0.072	−0.053	0.033	−0.286***	0.088
**Therapeutic Class: Reference NRTI**						
**NNRTI**	0.082	0.111	−0.211	0.181	0.336***	0.125
**PI**	0.854***	0.094	0.383***	0.112	1.672***	0.157
**FI**	1.981***	0.303	2.538***	0.300		
**Drug Age** ≥**5 Years = 1**	−0.256***	0.098	−0.274**	0.113	−0.428***	0.161
**Exclusive for<60 kg Patients = 1**	−1.256***	0.131	−1.093***	0.204	−0.928***	0.150
**Present in 1^st^-Line Therapy = 1**	−0.035	0.125	−0.013	0.117	0.217	0.170
**Number of Intraclass Substitutes**	0.017	0.030	0.058	0.051	−0.055	0.037
**Number of Potential Suppliers**	0.019	0.009	−0.009	0.022	0.049***	0.011
**Originator Drug = 1**	1.323***	0.109				
**Ln(GDP) ÷ 100,000**	1.202***	0.167	0.181	0.180	2.051***	0.207
**Number of Patients ÷ 10,000**	−0.099***	0.010	−0.086***	0.018	−0.124***	0.012
**Adjusted R^2^**	0.864		0.809		0.740	

*Significant at 10%;

**Significant at 5%;

***Significant at 1%.

Regarding drug characteristics, both therapeutic class and drug age hold significant price effects. In the case of originator drugs, whereas we observed no statistical difference between NNRTI and NRTI classes, everything else held equal, mean prices are 38% more expensive for PI (p = 0.001) and 254% for FI (p<0.001). For generics, NRTI mean prices increase 34% compared to NNRTI (p = 0.008) and 167% to PI (p<0.001). In terms of age, as expected, mean prices decrease as drugs pass the five-year threshold, the difference being nearly 27% for originator (p = 0.018) and 43% for generic drugs (p = 0.009). Moreover, drugs used exclusively by adult patients weighing less than 60 kg cost less in both originator and generic drugs (−1.093 and −0.928; p<0.001). Therapeutic guidelines, however, proved no significant association to price in either market segment.

As for market characteristics, only the generic segment seems responsive. *Ceteris paribus*, when demand increases 1% prices fall on average 0.29% (p = 0.001). Number of intraclass substitutes, however, shows no significant effect and number of potential suppliers, although significant, holds the opposite sign. In this generic segment, prices increase nearly twice the proportion of GDP increase (2.051%; p<0.001) and decrease 12% for every additional 10,000 patients treated. Originator drug prices vary only in response to number of treated patients (−9%; p<0.001).

Whereas segmented market analysis seems more adequate in modeling ARV price determinants in Brazil, unobserved heterogeneity could be interfering with some of our results. In-difference estimation, presented in Table 5, allows for in-depth analysis of the impact of dynamic aspects on price variation. On the side of originator drugs, time-varying characteristics provided no additional information. Statistical significance and mathematical signs remained the same as in Table 4. As for generic drugs, partial price elasticity to demand and GDP, as well as effect of number of treated patients on prices were confirmed. Additional information refers to the effect of therapeutic guideline changes and number of potential suppliers, where holding supplier constant reveals different findings from the previous estimation. In-difference estimation shows that the inclusion of a drug in recommendations for first-line therapy holds a downward effect on prices. Moreover, number of suppliers now shows the correct sign, indicating that the arrival (departure) of a supplier in the market forces prices down (up). The correct coefficient sign obtained through the in-difference estimation clearly reflects that the number of potential suppliers is a significant determinant of prices only when price changes are observed within the same firm. Indeed, the “wrong” sign associated to the variable number of potential suppliers in the pooled OLS estimation was probably due to the fact that such a model does not control for unobserved factors and was not able to take into account price variations within the same firm.

**Table 5:** Time-Varying Characteristics of the Brazilian ARV Market (1996–2009): In-difference Model.

Dependent Variable: ΔLn(PYD)	All		Originator Drugs		Generic Drugs	
	(n = 246)		(n = 78)		(n = 168)	
Changes between t and t+1	Coeff.	SE	Coeff.	SE	Coeff.	SE
**Δ Ln (QYD)**	−0.038**	0.016	−0.016	0.018	−0.051**	0.023
**Not in 1^st^-Line in ** ***t/*** **in 1^st^-Line in ** ***t+1*** ** = 1**	−0.044	0.057	0.048	0.060	−0.148*	0.086
**Δ Number of Intraclass Substitutes**	0.005	0.017	−0.029	0.023	0.027	0.021
**Δ Number of Potential Suppliers**	−0.034***	0.009	−0.013	0.020	−0.021**	0.010
**Δ Ln(GDP)**	0.680***	0.129	0.069	0.155	1.132***	0.173
**Δ Number of Treated Patients**	−0.140***	0.015	−0.112***	0.024	−0.158***	0.018
**Adjusted R^2^**	0.441		0.303		0.535	

## Discussion

Over the study period (1996–2009), mean ARV prices decreased in Brazil at a similar pace for both originator and generic drugs. In other words, both discounts from originator companies and generic competition have contributed to making ART more affordable in Brazil. Striking, however, is the fact that more than half of generic transactions in our dataset, considering the same ARV and supplier, correspond to price increase, whereas nearly 85% of originator transactions involve price decrease. These findings look rather paradoxical, but they corroborate those presented by Nunn and colleagues (2007) [23]. Using Brazilian ARV procurement data from 2001 to 2005, the authors concluded that while Brazil was able to obtain considerable discounts from originator companies, locally produced ARV prices remained higher than those of the global generic market and presented an increasing trend. One possible explanation is that originator drugs hold a higher margin from which to decrease prices, while generic suppliers operate already close to marginal cost. Another factor that cannot be ignored is that local currency underwent a near 35% appreciation against the US dollar between 2003 and 2009.

Further explanations can be found in relation to industrial, treatment and procurement policies carried out by the Brazilian government. As mentioned before, from 2002 onwards, the government prioritized public generic supply. This policy aimed at optimizing public sector production, which had started facing excess capacity [13]. In fact, real production costs of publicly supplied ARVs were not revised until 2007. Looking at nominal values from our database, prices in local currency did not change much since 2002 and it was not until 2008 that they started decreasing again. Our results, indeed, show an overall decreasing trend of demand for generic drugs, particularly in the most recent years, confirming previous findings by Grangeiro and colleagues (2006) [21] and Greco and Simao (2007) [22].

Another problem has to do with production scale. As Brazilian ART guidelines offer multiple regimen options instead of promoting systematic standardization of HAART, and some production processes require the use of exclusive industrial plant facilities, concomitant production of a high number of drugs shows rather limited efficiency. This is not the case of ARV production in the wider international market, where treatments tend to be standardized according to WHO guidelines and scale is much larger [30]. Finally, public laboratories lack vertical production capacity as they are not able to integrate the entire drug manufacturing process from API synthesis to drug formulation. Since public laboratories rely on external sources of API, whose supply is conditioned by public procurement legislation, and final ARV price paid by the Brazilian Ministry of Health are equalized across different public laboratories, production costs tend to be leveled-up. An extra profit margin sometimes becomes necessary in order to handle additional costs implied by risks of supply shortages and by raw-material purification procedures.

By stratifying the analysis according to originator and generic market segments, our modeling approach clarifies how differently originator and generic suppliers react to market size and competition in Brazil and highlights their distinct price dynamics. Originator drugs proved insensitive to various price determining factors, even after controlling for time-constant observed and unobserved heterogeneity. Given the close fit of originator and patented drugs (i.e., drugs holding either a valid or pending patent in Brazil; ρ = 0.852; p<0.001), this variable constitutes a proxy of patent protection, where no direct generic competition exists. Important exceptions in our sample refer to generic provision of Efavirenz since 2007 (under compulsory license), and, originator supply of Enteric Didanosine (off-patent) and Tenofovir (patent denied in 2008). Additionally, the degree of innovation seems to play an important role, as older drugs and older therapeutic classes tend to cost less. Although intraclass alternatives are available, this seems to produce no significant effect, suggesting that competition for originator drugs is limited by therapeutic restrictions and physician's prescription practices that may not systematically use opportunities of drug substitution. Finally, originator prices do not respond to bulk procurement, confirming Lucchini and colleagues' (2003) [26] hypothesis that monopsony (the existence of a single purchaser in a market) is able to compensate for monopoly power only when alternative suppliers are available.

Although external factors to the Brazilian market were not directly measured in our analysis, our results suggest pathways through which they may influence originator drug prices. We found no significant difference between NRTI and NNRTI prices in our sample, which are the only classes allowed in WHO first-line recommendations and where global generic competition and market scale are the highest. At the same time, although originator suppliers are more likely to benefit from changes in Brazilian therapeutic guidelines, as they progressively incorporate more patented drugs, the corresponding variable in our model estimation held no significant effect. Moreover, strategic pricing, as observed by Nunn and colleagues (2007) [23], dictates better discounts for drugs that, although patented in Brazil, have generic counterparts available in the global market. This practice may be related to the increased likelihood of compulsory licensing threats being made. Originator company strategic behavior may further explain why market size, as measured by number of ART-treated patients, holds a downward effect on prices.

The Brazilian ARV generic segment, on the contrary, resembles competitive markets. Prices react to demand volume, number of suppliers and economic cycles (as expressed in terms of GDP), as well as changes concerning therapeutic guidelines and number of treated patients that impact market scale. Price variation regarding drug age and therapeutic class may respond to marginal production cost variations [31]. Finally, price insensitivity to availability of alternative products, like in the originator segment, seems to refer to treatment characteristics that limit the degree of drug substitutability. These findings allow a better understanding of the consequences that changing market characteristics can hold on generic supply. Recent price increases may indeed reflect substantial drops in the number of available ARV suppliers, due to political choices that rendered this segment less competitive. As changes in therapeutic guidelines progressively shift demand towards the incorporation of patented drugs, the role of generic supply may become more and more limited. Although local generic production capacity proved essential to strengthen the bargaining power of Brazilian authorities in negotiations with originator companies, dependence on imported APIs may impair compulsory licensing of ARVs that are protected in the context of mandatory compliance to TRIPS by all developing countries, including India, after 2005.

### Study Limitations

Our study is not without limitations. Focusing the analysis in the Brazilian market, although improving data standardization, has reduced our sample size, especially in the case of originator drugs. However, it is reassuring that, for this market segment, results from pooled OLS and in-difference estimation support each other. Another limitation has to do with the extension of our findings to other developing countries given some Brazilian specificities, such as local production capacity, centralized procurement and low reliance on FDCs. Many low-income countries lack pharmaceutical laboratory facilities and count on third-parties for ARV procurement. Furthermore, the global generic ARV market has strongly specialized in FDC supply thanks to HIV care standardization promoted by the WHO. Therefore, the application of our model to other settings may require further adaptations, as for example to allow for the inclusion of FDCs where these represent an important share of ART, by employing relevant stratification techniques necessary to assure comparability between prices and purchased quantities. Moreover, due to availability of data and econometric modeling constraints, the variables we have used as proxies for capturing the amount of competition between firms in both segments of markets (number of intraclass substitutes and number of potential suppliers in the market) were rather crude; in the future, more sophisticated measures of market share by the largest firms may help improve the estimations.

### Final Remarks

Stratified analysis coupled by in-difference estimation, using ex-ante and ex-post information on price evolution, offers a more refined understanding of ARV price determinants and of how they may affect market dynamics. In Brazil, generic suppliers are more liable to respond to factors influencing demand size and market competition, while originator suppliers set prices strategically. These findings complement evidence provided by previous studies. Our analysis further demonstrates that changing characteristics in the generic segment, related to therapeutic, industrial and procurement policies, have been interfering in the process of ARV price formation in Brazil. While individualized care may improve treatment results and avoid costs related to premature regimen changes and toxicities, excessive complexity of ARV regimens can limit production efficiency. Our results highlight the importance of preserving generic market dynamics as it can also be supportive of ARV purchasers in price negotiations they undertake with originator companies.

Demand shift towards new and more potent drugs, as recommended by the latest WHO guidelines in a context of IPR strengthening, makes of the Brazilian experience a reference case for the challenges that may be faced by many developing countries as more PLWHA access HAART and live longer. In the presence of resource constraints, the sustainability of ART access clearly depends on further price decreases, especially for ARVs included in second and third-line regimens and for more powerful first-line therapies. Price discrimination (the differential pricing of a product for different markets) and IPR flexibilities, such as compulsory licensing, although they may help reduce prices, they may do so for a limited time-frame. Price discrimination is not consistently applied according to countries needs and income and relies on the good-will of originator companies [26]. Furthermore, compulsory licensing is highly dependent on available production capacity, including the local production of APIs where alternative sources are scarce or restricted, and requires strong political will to face retaliations from originator companies.

Our findings stress the need of preserving alternative sources of ARV supply. Therefore, the acquisition of vertical production capacity, the use of public procurement to create incentives for private sector participation, and, improvements in drug production efficiency remain important policy measures to increase countries bargaining power in price and voluntary license negotiations that can potentially benefit other health programs. Recently, a patent pool initiative has been put in place to incite originator companies issuing voluntary licenses to generic producers. The principle behind this proposal is that, while originator companies would earn additional royalties from drugs sold to treat a higher number of patients, generic producers would gain by entering the global market before patent expiry. The Brazilian experience suggests that potential disadvantages of patent pools, such as exclusivity agreements and risk of collusion, should be carefully monitored not to harm market competition.

## Supporting Information

Appendix S1 **List of Drugs Included in the Analysis.**
(DOC)

Appendix S2 **Comparable Dataset Characteristics.**
(DOC)

Appendix S3 **Endogeneity Detection and Correction.**
(DOC)
