# Supplementary material for: Modeling HIV/AIDS Drug Price Determinants in Brazil: Is Generic Competition a Myth?
Source: PLoS One. 2011 Aug 15;6(8):e23478. doi: 10.1371/journal.pone.0023478 (PMC3156239; doi:10.1371/journal.pone.0023478)
Supplement: Appendix S1 — List of Drugs Included in the Analysis. (DOC) [file pone.0023478.s001.doc]

The observations included in our analysis correspond to transactions on 21 ARVs in 27 different formulations: Abacavir (300mg), Amprenavir (150mg), Atazanavir (150, 200, 300mg), Darunavir (300mg), Delavirdine (100mg), Didanosine (100mg), Enteric Didanosine (250, 400mg), Efavirenz (200, 600mg), Enfuvirtide (90ml), Fosamprenavir (700mg), Indinavir (400mg), Laminudine (150mg), Lopinavir/Ritonavir (133.3/33.3, 200/50mg), Nelfinavir (250mg), Nevirapine (200mg), Raltegravir (400mg), Saquinavir (200mg), Stavudine (30, 40mg), Tenofovir (300mg), Zalcitabine (0.75mg) and Zidovudine (100mg).
