# Supplementary material for: Modeling HIV/AIDS Drug Price Determinants in Brazil: Is Generic Competition a Myth?
Source: PLoS One. 2011 Aug 15;6(8):e23478. doi: 10.1371/journal.pone.0023478 (PMC3156239; doi:10.1371/journal.pone.0023478)
Supplement: Appendix S2 — Comparable Dataset Characteristics. (DOC) [file pone.0023478.s002.doc]

Figure 1 and Table 6 provide information about the structure of the samples before and after data manipulation. The objective is to compare how similar the two datasets are in order to check if the manipulated dataset can adequately represent the original one. This can only be made by comparing the mean or proportions of time-constant variables. It is important to notice that all variables shown in Table 6 are binary, i.e., they indicate whether the characteristic is present (=1) or not (=0), and their corresponding values are presented in terms of sample proportions. The statistical test we then performed was that of the difference between proportions from the same variable in the two samples against the null hypothesis that these are similar. Indeed, the vast majority of these proportions are not statistically different from one another. Additionally, mean PYD in the original and manipulated datasets (1596.44 and 1355.05 USD, with standard deviations of 2578.148 and 2376.883, respectively) are not statistically different either.

**Figure 1. Relative Frequency Distribution of Observations in the Original and Manipulated Datasets.**

| **Table 6. Percentage Distribution of Time-Constant Characteristics in the Sample Before and After Data Manipulation** | | |
| --- | --- | --- |
|  | | |
|  | **Original Dataset** | **Manipulated Dataset** |
| **Variables** | **(n=378)** | **(n=246)** |
|  | **% of sample** | **% of sample** |
|  | | |
| **1996** | 1.6 |  |
| **1997** | 2.6 | 2.0 |
| **1998** | 6.3 | 2.4** |
| **1999** | 6.6 | 4.5 |
| **2000** | 7.4 | 6.1 |
| **2001** | 7.7 | 7.3 |
| **2002** | 7.1 | 9.3 |
| **2003** | 9.5 | 9.8 |
| **2004** | 9.3 | 11.4 |
| **2005** | 13.5 | 12.6 |
| **2006** | 8.2 | 12.6* |
| **2007** | 6.9 | 8.1 |
| **2008** | 8.2 | 7.7 |
| **2009** | 5.0 | 6.1 |
|  | | |
| **NRTI** | 60.3 | 63.8 |
| **NNRTI** | 12.7 | 11.0 |
| **PI** | 24.9 | 23.6 |
| **FI** | 1.6 | 1.6 |
| **II** | 0.5 |  |
|  | | |
| **Drug Age ≥ 5 years = 1** | 78.8 | 78.5 |
|  | | |
| **Patient Weight < 60kg = 1** | 7.1 | 7.7 |
|  | | |
| **Originator Drug = 1** | 33.3 | 31.7 |
|  | | |

Significant difference at: *10%; **5%; ***1% confidence level
