# Supplementary material for: Modeling HIV/AIDS Drug Price Determinants in Brazil: Is Generic Competition a Myth?
Source: PLoS One. 2011 Aug 15;6(8):e23478. doi: 10.1371/journal.pone.0023478 (PMC3156239; doi:10.1371/journal.pone.0023478)
Supplement: Appendix S3 — Endogeneity Detection and Correction. (DOC) [file pone.0023478.s003.doc]

We have implemented the method of augmented regression as suggested by Davidson and McKinnon (1993) [26]. We first addressed the problem of finding an adequate instrument by using ex-ante and ex-post row-information. The lagged variable, ln (QYD)_it_, available in the new configuration of our dataset, was employed. Ln(QYD)_it_ attends two main conditions as an instrumental variable:

1) There is a strong correlation between demand quantity in *t* and *t+1*, *i.e.*, the Pearson correlation between ln(QYD)_it_ and ln(QYD)_it+1_ is 0.726 (p<0.001);

2) The orthogonality between ln(QYD)_it_ and yearly dose prices ln(PYD)_t+1_ is confirmed by their non-significant correlation: -0.091 (p=0.119).

Next, ln(QYD)_it+1_ was regressed on explanatory variables and the instrument as follows:

(C.1) ;

where X is the set of explanatory variables used in the pooled OLS equation (equation 1 in the main text). Including $\ln(QYD)_{it}$ is a sufficient condition for instrumenting quantities in *t+1*. Next, residuals from this regression were included as an explanatory variable in the pooled OLS equation:

(C.2)

The objective was to test, on the one hand, whether the parameter
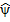
 is significantly different from zero. On the other hand, a global F-test comparing the fit of (C.2) with that of the main regression equation (without the estimated error term
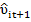
) was implemented to test the null hypothesis of exogeneity. The estimation of the equation (C.2) is presented in Table 7. The line in italic at the bottom of this Table represents residual estimates from the instrumental equation (C.1). The associated coefficients appear significant for the “all” regression (0.185; p=0.017) and the “generic drugs” regression (0.208; p=0.031). These results are consistent with the results from the F-tests (see Table 7) in favor of the endogeneity of QYD, whereas exogeneity is retained for the “originator drugs” case. Thus, for the whole sample and the generic drugs segment, the estimation of price determinants was effectuated by implementing the instrumental variables technique (two-stage least squares - 2SLS).

The problem of demand endogeneity in the in-difference framework is less constraining if we take into account that this technique controls two of the main endogeneity sources: unobserved heterogeneity and omitted variables. Although the main objective of the in-difference regression is to show whether controlling for unobserved heterogeneity reveals different patterns – in terms of price determinants – between the originator and the generic segments, its implementation sheds light on factors at the origin of demand endogeneity. Similar features of quantity effect on prices between the in-difference and pooled OLS estimations (after correcting for endogeneity where applicable), suggests that, in our sample, demand endogeneity is most likely caused by time-constant characteristics of the Brazilian ARV market, which are controlled for in the in-difference estimation.

| **Table 7. Endogeneity Test** | | | | | | |
| --- | --- | --- | --- | --- | --- | --- |
|  | | | | | | |
| **Dependent Variable : Ln(PYD)** | **All** | | **Originator Drugs** | | **Generic Drugs** | |
|  | **(n=246)** | | **(n=78)** | | **(n=168)** | |
| **Variables** | **Coeff.** | **ES** | **Coeff.** | **ES** | **Coeff.** | **ES** |
|  | | | | | | |
| **Intercept** | 7.584*** | 0.533 | 9.738*** | 0.748 | 6.476*** | 0.779 |
| **Ln(QYD)** | -0.235*** | 0.071 | -0.261* | 0.138 | -0.286*** | 0.087 |
| **Therapeutic Class: Reference NRTI** | | | | | | |
| **NNRTI** | 0.082 | 0.111 | -0.176 | 0.181 | 0.336*** | 0.124 |
| **PI** | 0.854*** | 0.094 | 0.339*** | 0.114 | 1.672*** | 0.155 |
| **FI** | 1.981*** | 0.301 | 2.209*** | 0.364 |  |  |
|  |  |  |  |  |  |  |
| **Drug Age ≥ 5 years = 1** | -0.256*** | 0.098 | -0.270** | 0.112 | -0.428*** | 0.159 |
| **Patient weight < 60kg = 1** | -1.265*** | 0.131 | -1.326*** | 0.251 | -0.928*** | 0.149 |
| **Present in 1st-Line Therapy = 1** | -0.035 | 0.124 | 0.244 | 0.202 | 0.217 | 0.168 |
| **Number of Intraclass Substitutes** | -0.017 | 0.029 | 0.071 | 0.051 | -0.055 | 0.036 |
| **Number of Potential Suppliers** | 0.009 | 0.009 | -0.009 | 0.022 | 0.049*** | 0.010 |
| **Originator Drug = 1** | 1.323*** | 0.109 |  | | | |
|  | | | | | | |
| **Ln(GDP) ÷ 100,000** | 1.202*** | 0.166 | 0.522* | 0.282 | 2.051*** | 0.205 |
| **Number of Patients ÷ 10,000** | -0.099*** | 0.010 | -0.084*** | 0.017 | -0.124*** | 0.012 |
|  | | | | | | |
| ***Estimated Residuals from Instrumenting Ln(QYD)*** | *0.185*** | *0.077* | *0.221* | *0.142* | *0.208*** | *0.096* |
|  | | | | | | |
| **Adjusted R²** | 0.866 | | 0.813 | | 0.745 | |
| ***F-test H_0_ : Ln(QYD) is exogenous*** | *5.73*** | | *2.43* | | *4.72*** | |
|  |  | |  | |  | |

*Significant at 10%; ** Significant at 5%; ***Significant at 1%
